# Analysis of Machine Learning Techniques Applied to Sensory Detection of Vehicles in Intelligent Crosswalks

**DOI:** 10.3390/s20216019

**Published:** 2020-10-23

**Authors:** José Manuel Lozano Domínguez, Faroq Al-Tam, Tomás de J. Mateo Sanguino, Noélia Correia

**Affiliations:** 1 Department of Electronic Engineering, Computer Systems and Automatics, University of Huelva, Av. de las Artes s/n, 21007 Huelva, Spain; jose.lozano@diesia.uhu.es; 2 Center for Electronic, Optoelectronic and Telecommunications, Faculty of Science and Technology, University of Algarve, 8005-139 Faro, Portugal; ftam@ualg.pt (F.A.-T.); ncorreia@ualg.pt (N.C.)

**Keywords:** smart road safety, pedestrian crossings accidents, vehicle detection, machine learning, time series forecasting

## Abstract

Improving road safety through artificial intelligence-based systems is now crucial turning smart cities into a reality. Under this highly relevant and extensive heading, an approach is proposed to improve vehicle detection in smart crosswalks using machine learning models. Contrarily to classic fuzzy classifiers, machine learning models do not require the readjustment of labels that depend on the location of the system and the road conditions. Several machine learning models were trained and tested using real traffic data taken from urban scenarios in both Portugal and Spain. These include random forest, time-series forecasting, multi-layer perceptron, support vector machine, and logistic regression models. A deep reinforcement learning agent, based on a state-of-the-art double-deep recurrent Q-network, is also designed and compared with the machine learning models just mentioned. Results show that the machine learning models can efficiently replace the classic fuzzy classifier.

## 1. Introduction

The so-called intelligent cities can now provide more responsible and efficient solutions to several problems, thanks to information and communication technologies (ICTs) and the many sources of information available [1]. Two fundamental facilitators of this new reality are wireless communication technologies and information analysis/treatment techniques [2] based on artificial intelligence (AI)—machine learning (ML) in particular—which will allow the era of “big data” to be embraced [3,4]. Its joint use is now enabling the development of processes that will transform sectors such as energy, economy, environment, health, and transportation, among others, into smart sectors [5].

An important area of intelligent cities is the intelligent transport system (ITS), which is a set of technological solutions designed to coordinate, improve, and increase transport safety on public roads [6]. Within this area, an intelligent road-marking system was first developed to reduce the rate of accidents around pedestrian crossings—a prior work from the same team [7,8]. The system has the ability to distinguish vehicles from pedestrians by means of a fuzzy classifier that performs sensor fusion using the following elements: (i) ultrasound sensors that detect people passing through the pedestrian crossing; (ii) magnetic field sensors to detect vehicles near a pedestrian crossing; and (iii) radar sensors to detect vehicles approaching the pedestrian crossing. As a disadvantage, the fuzzy labels need to be readjusted *n* times in each location where the system is placed, *n* being the number of system nodes per location.

Motivated by this drawback, the present article investigates the possibility of applying ML techniques to the detection of vehicles at pedestrian crossings. Unlike the previous work, this document does not cover techniques aimed at detecting pedestrians, which research work was already addressed in [9]. Applying these techniques would avoid the previously mentioned drawback, facilitating the installation of these systems. The ML techniques under analysis include classifiers, anomaly detectors, time-series forecasting (TSF), and deep reinforcement learning (DRL). More specifically, the contributions of this manuscript are the following: (i) to present a discussion on ML techniques used in literature for the detection of vehicles at pedestrian crossings; (ii) to go further and investigate the possibility of applying the long short-term memory (LSTM) and DRL techniques using a combination of sensors to detect vehicles at pedestrian crossings for the first time; (iii) to integrate LSTM with DRL to produce an even more robust data model than separately; (iv) to provide a general approach that aims to facilitate the installation of safe crosswalks; and last but not least, (v) to test our approach by assembling a new dataset, which has made available to the community. The proposed approach avoids the need for calibration, present in classic label-based fuzzy classifiers, and may also be suitable in systems that use magnetic sensors and are time-dependent, like intrusion detectors in security areas, traffic light controllers, overtaking assistance systems in autonomous vehicles, and vehicle detection in parking areas, among others.

The remainder of this article is organized as follows. Section 2 provides a discussion on work related to smart cities, and the ITS in particular. Section 3 describes the approach developed for vehicle detection, including the system description, problem formulation, and machine learning techniques used. Section 4 exposes the dataset structure, parameter settings, and experiments carried out, and shows the main results. Finally, Section 5 presents the conclusions as well as the future work.

## 2. Related Work

There are several AI- and ML-based solutions in the literature for ITSs that serve different purposes. In [10], for example, a vehicle detection system based on bioinspired algorithms and autonomic computing, and IBM’s MAPE-K is proposed to control the queues at traffic lights. In other applications, as in [11,12,13], historical data is used to detect accidents and generate alternative routes by combining radio frequency identification (RFID), 5G communication, and cloud services. These works use logistic regression (LR), multi-layer perceptron (MLP) neural networks, particle swarm optimization, adaptative boosting, and decision trees to release cognitive services over Microsoft Azure. Vehicle telemetry was also used to classify and detect abnormal situations on the roads (e.g., traffic jams) using a support vector machine (SVM) [14].

Another contribution of AI to turn cities into smart cities has been the monitoring of vehicles on public roads. Video surveillance systems have been widely used in this type of application, mainly because these systems are flexible and versatile, allowing the identification of movements and paths traced by vehicles [15]. Many papers describe solutions based on vision techniques and the AdaBoost learning algorithm due to their ability to detect and track vehicles in highly changing environments [16,17,18]. In [19], outdoor security cameras were integrated with a neural network classifier and the Mobilenet V1 SSD object detection model to detect and track vehicles, while in [20] a random forest (RF)-based method was proposed to detect vehicles under non-optimal lighting conditions. With the same intention, a system that subtracts the background from images and then detects vehicles using neural networks is described in [21]. Contrarily to the solutions based on cameras, the system proposed in [22] uses a 3D Laser Imaging Detection and Ranging (3D-LIDAR) sensor and a Deep Convolutional Neural Network (ConvNet) to detect vehicles under poor lightning conditions. Nevertheless, both vision-based and LIDAR sensor-based detection still have difficulties in detecting vehicles under adverse weather conditions (e.g., rain, fog, or snow). To improve detection, sensors can be used on the pavement to classify vehicles according to the vibration produced when they are moving [23]. The pattern in the time–frequency domain, generated by the sound of the vehicles when circulating, is used as source of information to detect targets [24].

AI techniques—and ML in particular—also enable the development of smart road safety (SRS) solutions for smart cities. In [25], for instance, a rear collision detection system for drivers was modelled using the vehicle acceleration and distance from the preceding car, and both random forest and neural networks were used. For the detection of vehicles moving in the wrong direction along highways, a camera-based system classifying the direction of the vehicle was proposed in [26], allowing traffic authority to be notified and act against such dangerous situations. Other solutions focus on avoiding damage to bikers. In [27], for example, the telemetry of a motorcycle was used to detect the roughness of the road and to determine the probability of an accident due to the state of the pavement. A method to detect the risk of an accident and activate airbags has been developed in [28] using accelerometer signals from the vehicle. The detection of pedestrians and animals on the road, to improve road safety, has also been studied. This is the case of systems designed to detect the intention to cross the road and alert drivers through cameras [29,30,31] and LIDAR technologies [32] applying dense, recurrent, or convolutional neural networks [33]. Other work focuses on the detection of pedestrians on zebra crossings using cameras and different classification schemes such as Haarcascade, histogram of oriented gradients (HOG), single-shot detector (SSD), and you-only-look-once (YOLO) [34]. The detection of stopped or transiting animals on the road was also studied in [35] for crashes to be avoided. Vision techniques, k-nearest neighbors (KNN), and random forest were used.

Table A1 (Appendix A) summarizes the main features of the previously described state-of-the-art proposals, together with our proposal for comparison. In general, most of the approaches to detecting targets are based on optical cameras or LIDAR sensors. This makes the systems vulnerable to poor lightning scenarios or adverse weather conditions. In addition, these are mainly on-board systems and their actions are limited to the presence of vehicles on the public roads. In contrast, the system proposed in this manuscript will be permanently located on streets so that vehicles are detected, and road safety is improved. Furthermore, the proposed approach is robust against low visibility and bad weather conditions due to the type of sensors used. Besides analyzing LR, RF, MLP, and SVM techniques also used by other authors although in different contexts, this article goes further and investigates the possibility of applying LSTM and DRL techniques for the first time. The reasons behind including the last two techniques are the following: (i) time-series forecasting in LSTM uses not only the immediate observation from the sensors but also the historical observations to produce coherent visual alerts; (ii) LSTM and DRL can be integrated (i.e., LSTM–DRL) to produce an even more robust model; (iii) unlike other classifiers, LSTM–DRL can be extended for online training.

## 3. Approach Description

### 3.1. System Description

**Figure 1:**
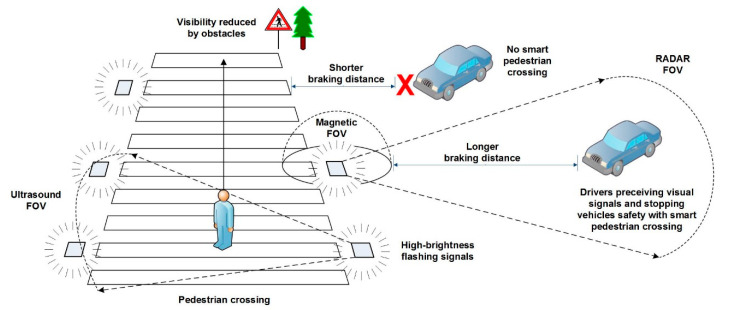
General description of the system and field of view for each sensor.

The system previously developed by the authors was aimed at reducing road accidents. Initially, the way to interact with the environment was to distinguish pedestrians from vehicles on zebra crossings, so that visual alerts could be generated. The system controller was implemented using a fuzzy classifier whose logic performed sensory fusion, combining data from different sensors. A general overview of the system, along with the orientation and field of view (FOV) of each sensor, is depicted in Figure 1. The ultrasound sensor measures the distance at which an obstacle is from the pedestrian crossing, offering a range from 60 cm to 5 m. The SRF485WPR sensor (Robot Electronics, Attleborough, UK) is the one used in this implementation. The magnetic sensor measures the Earth’s magnetic field for the X, Y, and Z axes, whose values are altered when a vehicle approaches or stops over a pedestrian crossing. The sensor used in this implementation is the LIS3MDL (STMicroelectronics, L’Hospitalet de Llobregat, Spain). The RADAR sensor provides measurements of the speed and signal strength reflected by an object, the latter being very useful to determine the volume of the target. The HB100 (AgilSense, Ang Mo Kio, Singapore) is the model used for implementation. Further technical details on the sensors used in this implementation can be found in [7,8].

### 3.2. Problem Formulation

The previously fuzzy classifier achieved an accuracy of 96.64% and a precision of 100% after calibrating the system for a specific geographical place. Nevertheless, this classifier has the disadvantage of requiring a readjustment of labels according to its physical location on the public road, as mentioned in Section 1. More precisely, the labels used were the following: (a) “Near”, “Medium”, and “Far”, for the ultrasound sensor to indicate how far is a target; (b) “Near-negative”, “Far”, and “Near-positive”, for the magnetic sensor to indicate, along the XYZ axes, if there is a vehicle stopped or approaching a pedestrian crossing; (c) “Low” and “High”, for the RADAR sensor to denote the presence of a target based on its reflected signal, together with “Minor” and “Major” for the RADAR to indicate whether the target approaches slowly or quickly. Since a magnetic field can be altered by the presence of power cables, pipes, and other ferromagnetic elements, often present on roads, the magnetic sensor labels must be readjusted according to the location of the system, which is a drawback.

### 3.3. Methodology

The following workflow is required for the development of the proposed AI-based approach: (1) a data collection and labeling procedure is carried out that takes into account diversity when collecting data (i.e., collection at different places and times); (2) the resulting dataset is analyzed and preprocessed to make it ready for ML models, keeping in mind that the dataset was intrinsically imbalanced, and that some models require time dependency between samples, among other requirements; (3) a set of AI-based models are applied to vehicle detection, the ones considered most appropriate for vehicle detection; (4) a DRL agent based on a state-of-the-art double-deep recurrent Q-network (DDRQN) algorithm is designed; (5) a set of experiments that are appropriate to the problem under study are outlined; (6) a statistical analysis including area under the curve (AUC) metrics and receiver operating characteristic (ROC) curves is performed, for model comparison. As far as it is known, the just-mentioned type of DRL agent and statistical analysis have not been previously applied to vehicle detection.

### 3.4. Machine Learning Techniques Used

To avoid the drawback of requiring recalibration of fuzzy labels, in this article we take a step forward and improve results with a unique detector for several places using ML techniques. The problem is treated as binary classification, anomaly detection, and even time-series prediction. Moreover, DRL is also used—even as an unsupervised method—assuming a reward function with a binary classification nature. Therefore, the techniques used were organized into classifiers, anomaly detectors, time-series forecasting, and DRL.

**Figure 2:**
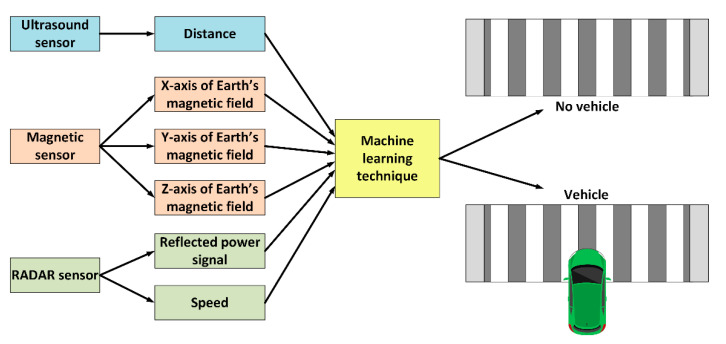
Machine learning approach implemented in the system.

The reasons for choosing the previously mentioned models are: (i) from each category of techniques we have chosen an approach. For instance, logistic regression was chosen among linear classifiers. Other alternatives are possible, like Fisher’s linear discriminant (FLD). However, these have the same nature and choosing one of them is appropriate. Moreover, (ii) the problem can be better understood if tackled in different ways, allowing a multi-unit seamless model to be developed in the future, which may require a new hardware setting to orchestrate multiple devices at the same time. The approach used to implement the ML models, and determine their performance, is shown in Figure 2. The yellow block is to be replaced by one of the techniques described in the next subsections.

#### 3.4.1. Classifiers

The classifiers used are logistic regression, random forest, and multilayer perceptron, which are all widely known supervised methods. These classifiers were all implemented with the scikit-learn library of Python [36]. The first one, the logistic regression, was designed to detect two different classes and the L2 norm was used to control the overfitting. This method is easy to implement and effective because it does not require data scaling. However, it can easily fall short to handle non-linear mappings [37].

The second classifier, random forest, was configured with 15 estimators. Random forest offers the advantage of handling large amounts of data and does not suffer from overfitting. On the other hand, it is slow to predict and hard to interpret how it performs [37].

Finally, the last classifier is the multi-layer perceptron, which was optimized with the Adam optimizer. The multi-layer perceptron consists of 4 hidden layers, each with 100 neurons, and 20% of the training data was used for validation to ensure the stability of the model. This has the capability to learn and model non-linear complex relationships, as well as to make predictions using data that have not been previously visited. On the other hand, it can be difficult to tune its hyperparameters and it may require long training for large neural networks [37].

#### 3.4.2. Anomaly Detector

The anomaly detector utilized is the one-class support vector machine (one-class SVM), which is an unsupervised outlier detector. This method was also implemented in Python with scikit-learn. The one-class SVM allows the detection of outliers (or anomalies) in the data. The use of this technique allows us to treat vehicles as outliers and the “No vehicle” state (i.e., the most frequent state of pedestrian crossings) as the “normal” state. The uniqueness of this method, when compared to the others, is that it only needs to be trained with the “No vehicle” class data. It has the advantage of offering a good handling of imbalanced classes, it only needs to train instances of the target class and it is very sensitive to outliers. On the other hand, it requires the right selection of hyperparameters and kernels [38].

#### 3.4.3. Time-Series Forecasting

**Figure 3:**
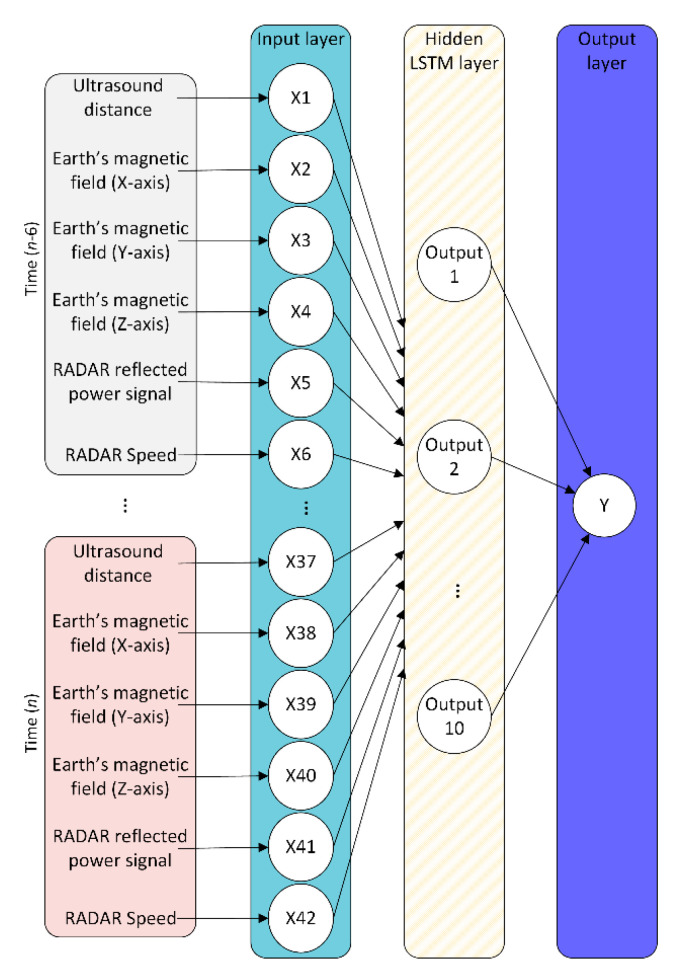
Long short-term memory (LSTM)-based neural network structure.

To avoid the system generating inconsistent visual alerts, it is necessary not only to consider the current measurements from sensors but also the last few ones. This way, the output of the system will depend on a sequence of observations. This means that the vehicle detection problem can be treated as time-series forecasting, allowing the use of an LSTM architecture. LSTM networks are an important piece in modern time-series forecasting and to sequence deep learning models. The reason for using an LSTM instead of others (e.g., statistical autoregressive models) is that LSTM networks can be integrated into DRL architectures, as explained below. An LSTM is a recurrent neural network (RNN) equipped with gated cells able to remember important information and forget the irrelevant. An LSTM converts the input sequence into memory and hidden states. These can be used to forecast future data based on some training samples, and it offers better long-term modeling and a more robust vanishing gradient than conventional RNN. However, an LSTM requires more computational and memory capacity due to the multiple memory cells [39]. As shown in Figure 3, the used LSTM-based neural network architecture consists of a total of 42 input neurons, corresponding to the number of time instants and different sensor measurements for each time instant. Then, the LSTM layer is made up of 10 units that are fully connected to the input layer. Finally, the output layer is made of a single neuron completely connected to the LSTM layer, being this neuron responsible for predicting whether a vehicle is—or is not—currently over a crosswalk. The neural network structure has been determined following the instructions described in the Parameter Settings section.

#### 3.4.4. Deep Reinforcement Learning

DRL is an emergent learning scheme whereby an agent learns by interacting with the environment. It is an unsupervised method and can be trained online. Here in this work it is trained offline for it to be compared with the other well-established methods. An online training system will be explored in the future.

The agent (usually represented as a deep neural network for efficiency purposes) observes a state from the environment and performs actions. According to the performed action, it receives a reward and a new state. The agent’s objective is to maximize the accumulated future reward. Therefore, training an agent means to find a policy that will map states to actions such that the accumulated reward received by the agent is maximized. In this work, the DDRQN model is used [40]. DDRQN uses an LSTM layer (i.e., LSTM–DRL) to obtain time relevance between consecutive states. This allows to reduce the overestimations and the number of operations to calculate them [41]. Therefore, the agent developed in this work uses the current and historical data in its state to recognize time-related events. The reward function is as simple as 1 if the agent selects the right class, and −1 otherwise. This rewards the agent for correct selections and penalizes it for wrong ones. The action space of the agent is {“No vehicle”, “Vehicle”}, where the LSTM–DRL agent decides if there is a vehicle approaching the crosswalk, or not, according to the received sequence of observations from the sensors.

**Figure 4:**
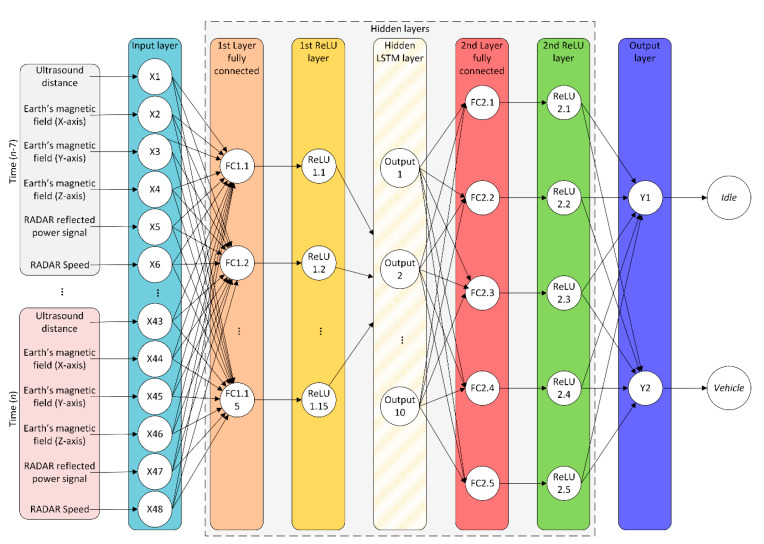
Neural network structure for the DRL used.

As shown in Figure 4, the neural network architecture used in the DRL model has a total of 48 input neurons corresponding to the measurements of the different sensors of the system according to each instant of time (*n*), including historical data (up to *n*-7). The hidden layers of the neural network are initially made up of a fully connected layer of 15 neurons, followed by an activation layer where the Rectified Linear Units (ReLU) function is used as activator. At the output of this layer, an LSTM layer with 10 units is completely connected with the previous layer. This output is linked to a second fully connected layer of five neurons. The output of this layer is connected to a second ReLU activation layer. Then, two neurons (Y1 and Y2) fully connected to the previous layer are used. The neural network structure has been determined following the instructions described in the Parameter Settings section.

## 4. Experimentation

### 4.1. Dataset Structure

The proposed approach uses data generated by three different sensors as predictor variables. Table 1 shows such set of predictor variables and range values. The data are used by the models to identify whether there is, or is not, a vehicle circulating through the intelligent pedestrian crossing.

For this purpose, two classes are used: “Vehicle” and “No vehicle”, represented as 1 and 0, respectively. These classes are used as ground truth (GT). Measurements from sensors were normalized using the Min–Max method for an adequate performance of the ML algorithms [42]. Normalization values are also shown in Table 1.

The dataset used to train the ML models, for these to determine whether a vehicle is approaching a pedestrian crossing or not, was collected from a real environment and is available at http://www.uhu.es/tomas.mateo/investigacion/dataset.zip. This dataset includes a total of 86,960 labeled tuples. The dataset is imbalanced: the “No vehicle” class has a total of 80,915 tuples (93.05%); whereas the “Vehicle” class has a total of 6045 tuples (6.95%). To balance the dataset, the NearMiss subsampling technique was used [43]. The balanced dataset ends up having 6362 tuples for the “No vehicle” class and 6045 tuples for the “Vehicle” class, which corresponds to 51.28% and 48.72%, respectively. Therefore, the size of the dataset used at the classification stage consists of 12,407 labeled tuples. The dataset is ordered by a tuple index to keep time linearity, which is necessary for some ML models to work properly.

The data collection process has been carried out in several real environments under fluid traffic conditions. The devices were placed on the roadway near the line of the pedestrian crossing, while the vehicles passed over devices—or on their sides—with an average speed of 18.20 ± 26.16 Km/h. This paper focuses on the detection of vehicles only as the basis to study the feasibility of using ML techniques instead of a fuzzy logic approach. So, the data collection procedure consisted of monitoring the system’s interactions with vehicles and environment, and then storing the data with both vehicles and no vehicles circulating. Because of this, cases involving pedestrians were filtered out and no data was recorded. Tests were performed both in Portugal and Spain, more specifically in the urban areas of Gambelas (Faro) and Bollullos Par del Condado (Huelva). Four points were in the University of Algarve, two points were in Rua Manuel Gomes Guerreiro, one point was in Rua Comandante Sebastião da Costa, one point was in Praceta Orlando Sena Rodriguez, and another point was in Sector Pp1 Cruz de Montañina. These locations present different terrestrial magnetic fields either due to their nature or to different elements found on public roads (e.g., traffic signs and streetlights among other ferromagnetic elements). To illustrate the different magnetic field values at these locations, Table 2 shows the average values and standard deviations for the X, Y, and Z axes of the magnetic sensor for both circulating and non-circulating vehicles. On the one hand, the table shows that there is a small difference between the values for “Vehicle” and “No vehicle” conditions at each location (i.e., a minimum of 0.0051% and maximum of 0.6532%), which is in practice very difficult to calibrate. On the other hand, the average values for the magnetic field sensor have no correspondence between sites with very similar values (e.g., *X*-axis for the column “No vehicle” of Praceta Orlando vs. the *X*-axis for the column “Vehicle” of Cruz Montañina). This means that a calibration process at one site is unrelated to another, being necessary to start a new labeling procedure of the system to differentiate circulating and non-circulating vehicles. The complexity of generating a single vehicle-classifier for multiple locations elevates to *N* × *L*, *N* being the number of nodes of the intelligent pedestrian crossing and *L* the number of locations. Details on the weather conditions for which the data were collected as well as the duration of each data collection process are given in Table 3. Finally, the system uses wireless communication to collect the data from the sensors. A portable access point (AP), a personal computer with WampServer software, and hypertext transfer protocol (HTTP) were used. The WampServer software has the capability to handle HTTP requests from the nodes (through an Apache server) and store the data in a MySQL database. The collected tuples were manually labelled as “No vehicle” or “Vehicle”.

### 4.2. Parameter Settings

The hyperparameter tuning of LR, RF, MLP, and one-class SVM methods has been carried out using the Grid Search Cross Validation technique implemented in scikit-learn [36], while the tuning of the LSTM and the DRL methods has been empirically determined. The method used to determine the best fit for LSTM and DRL is as follows. A combination matrix of the hyperparameter values is generated, the models undergo learning with these hyperparameters using cross-validation to get the metrics for that learning model, and these metrics are added to the combination matrix. The metrics used in this process have been the accuracy and, in case of a tie, the true positive rate and then the false positive rate. Once the metrics of the first iteration are compared, the best metrics are selected and, therefore, their hyperparameters. Then, a new matrix is established with values close to the best hyperparameters as well as a linear distribution of the values among the best hyperparameters. This process continues iteratively and ends when similar metrics are reached in all cases. The values adopted for each method are shown in Table 4.

### 4.3. Results and Discussion

The experiments carried out consisted of submitting the balanced data to the techniques described in the previous section. The cross-validation approach was used for this purpose [44]. The 12,407 tuples, resulting from the preprocessing and data balancing process, were divided into five folds, each one having a total of 2480 tuples. Among them, four folds were used for training whilst one was used for testing. The splitting of the dataset was performed to keep the required temporal linearity. The class distribution of the dataset, according to the folds, is shown in Table 5.

To determine the efficiency of each algorithm, the ROC analysis [45] was used (i.e., sensitivity of each algorithm to detect vehicles). The performance was obtained from a confusion matrix of 2 × 2 elements that relates positive (*p*) and negative (*n*) outcomes (Table 6). A vehicle detection is considered positive whilst the detection of the “No vehicle” class is considered negative.

**Figure 5:**
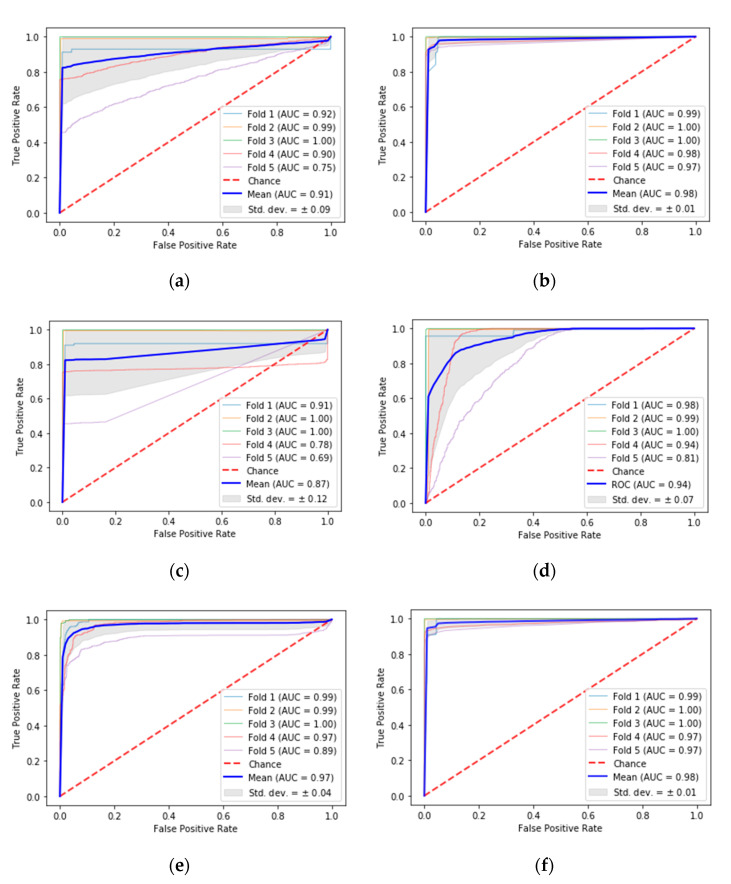
ROC analysis: (**a**) logistic regression; (**b**) random forest; (**c**) multi-layer perceptron; (**d**) one-class SVM; (**e**) long short-term memory; (**f**) deep reinforcement learning.

After training the models, taking into account the previous considerations, these were subjected to tests with data not previously used in the training phase. The model that best detected the vehicles was random forest, which achieved a true positive rate (TPR) of 96.82%, false positive rate (FPR) of 1.73%, precision of 98.63%, F1 of 97.68%, accuracy (ACC) of 97.85%, and AUC of 0.98. This can be considered as excellent in a scale of [0.97, 1), as argued in [46]. One drawback of RF is that its decisions are difficult to interpret. The following best-performing models are those that consider the time dimension of the data. These were the deep reinforcement learning (TPR = 92.94%, FPR = 3.73%, precision = 95.00%, F1 = 93.70%, ACC = 94.51%, and AUC = 0.98) and LSTM (TPR = 92.60%, FPR = 5.07%, precision = 95.14%, F1 = 93.18%, ACC = 93.83%, and AUC =0.97), both considered as excellent. The anomaly detector model, one-class SVM, also offers very good results—although lower than the previous models—achieving a TPR of 93.38%, FPR of 15.59%, ACC of 92.08%, and AUC of 0.94. Finally, the multi-layer perceptron and logistic regression had a performance that can be considered as good. Nevertheless, their accuracy rate is reduced when compared to the other models. A summary of these results is shown in Table 7, while the ROC curves for each of the ML techniques are shown in Figure 5. The blue color represents the average ROC curve, the gray shaded area shows the standard deviation, and the soft-blue, orange, green, pink, and purple colors stand for each of the folds used to test the models. Table 7 also includes the performance achieved with the fuzzy classifier used previously. This classifier provided an excellent performance as it was calibrated for a specific location prior to testing. Despite a slight performance drop, the proposed machine learning approaches allow generalization of vehicle detection without the need to calibrate the system.

The overall results are summarized as follows. Firstly, LR offers a good result but is not as outstanding as other techniques. The LR reaches an accuracy of 90.84%; its performance is good, but its accuracy, TPR, and ACC are easily surpassed by other models. Its FPR is, however, the lowest of all the techniques used. Additionally, this model is not very reliable because it presents a high standard deviation for the TPR and ACC. Secondly, RF offers the best success rate from all the models, complying with the theory of its ability to optimize the accuracy. Moreover, this model is very stable as it achieves a low standard deviation in all measurements. Thirdly, the model based on MLP offers good results (ACC of 90.98%), but it is not reliable due to the high standard deviation of the TPR and the ACC. Fourthly, the one-class SVM is the least reliable of all the methods because it presents the highest FPR and the highest standard deviation. Fifthly, LSTM offers excellent and stable results, as expected in a theorical way, since it considers the time series. In this regard, the vehicle detection depends largely on the time because two representations of the crosswalk state can be similar for different situations in different locations. Finally, DRL also offers excellent and stable results due to the construction of a specialized agent. The input structure based on current and historical data allows this method to identify the temporal events generated by vehicles when approaching a pedestrian crossing.

In addition to the previous analysis, the AUC was studied to empirically determine the performance of each model used. The results obtained confirm the outcomes previously exposed, being that the models based on RF and DRL are the best techniques, with an AUC of 0.98 and a standard deviation of 0.01 each. That is, they have similar separability of classes, which can be confirmed by the shape of their ROC curves in Figure 5. These were followed by LSTM, which achieved an AUC of 0.97 and a standard deviation of 0.04. The one-class SVM offered a very good performance, reaching an AUC value of 0.94 and a standard deviation of 0.07. Finally, the methods based on LR and MLP presented the lowest performance, which is in line with the AUC results of 0.91 and 0.87, respectively.

From all the results exposed it is possible to state that ML techniques are an adequate solution to detect vehicles near pedestrian crossings, and a better choice than the fuzzy classifiers used in our previous work. Although the results obtained with the ML techniques offer a lower performance than those achieved with the fuzzy classifier calibrated for a specific site, the experimentation carried out demonstrate their feasibility to replace the fuzzy classifier. These techniques allow us to place the system in any location without it needing to be calibrated, with a very good or excellent performance. The most effective and reliable ML methods were RF and LSTM–DRL due to their high performance and stability, as shown in Table 7 and Figure 5. On the contrary, the least-recommended methods to detect vehicles, under this system, were LR, MLP, and one-class SVM in particular. LR and MLP are least-recommended due to the high deviations present in their TPR and ACC, and one-class SVM is least-recommended due to its high FPR and standard deviation. In general, the temporal recognizing methods, like DRL and LSTM, offer better performance than the other methods, except for RF. From another perspective, the datasets can be seen as having a limitation: the locations used for data collection were always around the 37th parallel.

This fact, however, does not prevent us from confirming that the models under analysis are capable of replacing the classic fuzzy calibration method. Furthermore, the current dataset—although being representative and including captures of cars, motorcycles and buses—does not reflect all types of possible vehicles that can circulate on public roads (e.g., bicycles, electric scooters, trucks or vans, among others). This lack of samples in the current dataset may have limited the performance of the system for these kinds of targets.

## 5. Conclusions

Thanks to the support of ICTs, smart cities are becoming a reality and can now improve or create new services that enhance the lives of their inhabitants. Intelligent transport systems and intelligent road safety are among those services in which a significant amount of innovation has emerged. This paper contributes to such field by investigating how different machine learning techniques can be applied—as an alternative to classic fuzzy logic—to generalize vehicle detection using sensor data from intelligent pedestrian crossings. To achieve this goal, several machine learning techniques were implemented in our system and their performance was evaluated. The methods were divided into classifiers (i.e., LR, RF, and MLP), anomaly detector (i.e., one-class SVM), time-series forecasting (i.e., LSTM) and DRL.

The training and testing of the different techniques were carried out with real data collected from a total of five different locations in both Portugal and Spain, under fluid traffic conditions. The dataset created from tests performed in real environments has been assembled and made public for the community. Training and validation data were obtained using a cross-validation scheme with five folds. The best results were obtained by the RF model, having a TPR of 96.82%, FPR of 1.73%, ACC of 97.85%, and AUC of 0.98. The next best results were obtained by DRL and LSTM models, with high ACC (94.83% and 93.83%, respectively) and high AUC (0.98 and 0.97 respectively). In contrast, LR and MLP offered the least reliable performance from all methods due to the low AUC obtained (0.91 and 0.87, respectively). Therefore, the results suggest that it is feasible to use RF, DRL, and LSTM—which present similar metrics—to replace the fuzzy logic approach for vehicle detection.

Future work will focus on real-time vehicle detection deployment, using machine learning techniques, with infotainment purposes. This could allow the system to be used as a traffic monitoring device, being able to count the number of vehicles circulating and to record when these detections take place. In addition, more samples will be collected in real environments, with a broader spectrum of vehicles (i.e., not only cars, motorcycles, and buses but also bicycles, scooters, trucks, or vans), creating more robust and generalized machine learning models. In this sense, samples should also be taken considering other locations (e.g., mountain areas and latitudes different from the 37th parallel) and at different times of the day (e.g., at the sunrise or sunset). This way, models—especially those that detect changes or patterns in time series—are expected to improve their reliability even more. This study can be generalized to applications studying a phenomenon that changes over time, as well as those that may occur in different locations (e.g., detection of intruders, overtaking or avoiding obstacles in autonomous vehicles, or vehicle detection in parking lots).

## Figures and Tables

**Table 1:** Sensor measurement ranges and corresponding normalization.

Measurement	Range Value	Normalized Value
Ultrasound distance	60–500 cm	0–1
Magnetic *X*-axis	−32.768–32.767 Gauss	0–1
Magnetic *Y*-axis	−32.768–32.767 Gauss	0–1
Magnetic *Z*-axis	−32.768–32.767 Gauss	0–1
Reflected signal power RADAR	0–1 n/a	0–1
Speed RADAR	0–100 Km/h	0–100
Ground truth	0–1	0–1

**Table 2:** Normalized average magnetic sensor measurements by location, axis and vehicle presence.

	No Vehicle			Vehicle		
Calibration Site	*X*-axis (avg. ± dev.)	*Y*-axis (avg. ± dev.).	*Z*-axis (avg. ± dev.)	*X*-axis (avg. ± dev.)	*Y*-axis (avg. ± dev.).	*Z*-axis (avg. ± dev.)
Campus 1	0.499 ± 0.000	0.4580 ± 0.001	0.581 ± 0.001	0.493 ± 0.009	0.461 ± 0.009	0.587 ± 0.012
Campus 2	0.510 ± 0.06	0.500 ± 0.023	0.582 ± 0.01	0.504 ± 0.027	0.502 ± 0.032	0.586 ± 0.031
Campus 3	0.501 ± 0.001	0.455 ± 0.004	0.582 ± 0.001	0.499 ± 0.006	0.455 ± 0.008	0.587 ± 0.010
Campus 4	0.495 ± 0.000	0.458 ± 0.000	0.581 ± 0.001	0.490 ± 0.008	0.460 ± 0.010	0.586 ± 0.012
Manuel Gomes Guerriro	0.513 ± 0.001	0.513 ±0.001	0.584 ± 0.001	0.509 ± 0.010	0.514 ± 0.007	0.589 ± 0.009
Praceta Orlando Sena Rodriguez	0.480 ± 0.001	0.4803 ± 0.001	0.587 ± 0.001	0.476 ± 0.007	0.481 ± 0.009	0.592 ± 0.010
Comandante Sabastião da Costa	0.511 ± 0.000	0.456 ± 0.000	0.584 ± 0.000	0.504 ± 0.005	0.458 ± 0.010	0.586 ± 0.013
Cruz Montañina	0.478 ± 0.001	0.492 ± 0.001	0.585 ± 0.001	0.479 ± 0.006	0.492 ± 0.007	0.587 ± 0.010

**Table 3:** Weather and temporal conditions of data collection.

Site	Temperature (°C)	Humidity (%)	Weather	Date	Day Time	Duration (min)
Campus 1	24	57	Partly cloudy	25 July 2019	19:34	22
Campus 2	23	62	Sunny	5 August 2019	19:34	39
Campus 3	24	83	Partly Sunny	9 August 2019	13:37	18
Campus 4	24	83	Sunny	23 August 2019	10:36	29
Manuel Gomes Guerriro	25	58	Partly cloudy	4 September 2019	10:37	23
Praceta Orlando Sena Rodriguez	26	59	Partly cloudy	4 September 2019	11:22	25
Comandante Sabastião da Costa	25	68	Partly cloudy	4 September 2019	19:38	18
Cruz Montañina	21	67	Partly cloudy	9 September 2019	10:32	77

**Table 4:** Optimized parameters for machine learning (ML) models.

Technique	Variable	Value
Logistic regression	C	0.09
	Penalty	L2
	Random state	1
	Solver	Newton-cg
Random forest	Random state	1
	Number estimators	15
Multi-layer perceptron	Solver	Adam
	Hidden layers	4
	Learning rate	0.0001
	Hidden layer neurons	100
	Maximum iterations	1000
	Validation fraction	20%
One-class support vector machine	Nu	0.01
	Gamma	0.77
	Kernel	Radial basis function (RBF)
Long short-term memory	Number of time instants	7
	Intermediate layer	10
	Output layer neurons	1
	Optimizer	Adam
	Learning rate	0.0005
	Epochs	20
	Validation fraction	20%
Deep reinforcement learning (RDDQN)	Historical length	8
	Episodes	100
	Steps	2470
	Neurons of first layer fully connected	15
	Neurons of LSTM layer	10
	Neurons of second layer fully connected	5
	Neurons output layer	2
	Learning rate	0.0001

**Table 5:** Distribution of entries in “No vehicle” and “Vehicle” classes.

Fold	Class “No Vehicle”	Class “Vehicle”
1	680	1800
2	1965	515
3	1111	1369
4	1254	1226
5	1350	1130

**Table 6:** Confusion matrix for the receiver operating characteristic (ROC) analysis.

	Real Value		Total
	*P*	*N*	
Prediction			
*p′*	True positives (TP)	False positive (FP)	*P′*
*n′*	False negatives (FN)	True negatives (TN)	*N′*
Total	P	N	

**Table 7:** Results for the machine learning models.

Technique	TPR (%)	FPR (%)	P (%)	A (B/W) (%)	F1	AUC
LR	82.02 ± 22.85	0.22 ± 0.37	99.77 ± 0.25	91.08 (99.96/75.00)	0.88 ± 0.16	0.91 ± 0.09
RF	96.82 ± 4.05	1.73 ± 1.69	98.63 ± 0.53	97.85 (99.60/95.32)	0.98 ± 0.02	0.98 ± 0.01
MLP	82.11 ± 22.88	0.93 ± 1.95	99.77 ± 0.25	90.98 (99.96/75.00)	0.88 ± 0.16	0.87 ± 0.12
One-class SVM	98.38 ± 2.24	15.59 ± 23.19	89.91 ± 14.27	92.08 (99.96/75.44)	0.93 ± 0.08	0.94 ± 0.07
LSTM	92.60 ± 8.47	5.07 ± 3.26	95.14 ± 2.76	93.83 (99.02/86.78)	0.93 ± 0.06	0.97 ± 0.04
DRL	92.94 ± 9.92	3.73 ± 3.71	95.00 ± 4.37	94.51 (99.80/87.05)	0.94 ± 0.06	0.98 ± 0.01
Fuzzy logic	100	0	100	100	1	N/A

TPR: true positive rate; FPR: false positive rate; P: precision; A: accuracy with the best (B) and worst (W) values; F1 score in range [0, 1]; and area under the curve (AUC) in range [0, 1]. All measurements include the standard deviation except for the fuzzy logic technique and the accuracy.

## Appendix A

**Table A1:** Features of related work discussed in Section 2.

Reference	Field of Application	Target to Detect	Sensing Device	AI Technique	Metric Used	Year
[7,8]	SRS	Pedestrians & vehicles	Ultrasound; magnetic field; RADAR	Fuzzy logic	ACC; TPR; FPR; Precision; AUC	2018; 2018
[10]	ITS	Traffic jams	Simulated	Bio-inspired algorithm; autonomic computing	Queue of vehicles in a traffic light	2015
[11]	ITS	Traffic jams	5G; RFID	Microsoft Azure cognitive services	N/A	2019
[12]	ITS	Traffic jams	Smart phone	LR; bagging; AdaBoost; voting; trivial	ACC	2017
[13]	ITS	Traffic jams	Camera; security software	MLP; particle swarm	Free flow traffic	2019
[14]	ITS	Traffic jams	Telemetry	One-class SVM; logit	ACC: Recall; Precision	2018
[16]	ITS	Vehicles	Camera	AdaBoost	TPR; FDR	2014
[17]	ITS	Vehicles	Camera	AdaBoost	Detection time	2019
[18]	ITS	Vehicles	Camera	AdaBoost	TPR; FPR	2019
[19]	ITS	Vehicles	Camera	SSD MobileNet V1 model	ACC	2019
[20]	ITS	Vehicles	Camera	RF	N/A	2019
[21]	ITS	Vehicles	Camera	Naive Bayes; KNN; ANN	ACC	2019
[22]	ITS	Vehicles	3D-LIDAR	ConvNet	ACC	2018
[23]	ITS	Vehicles	Vibration	Naive Bayes; RBFN; SVM; MLP	TPR; FPR; Precision; Recall	2016
[24]	ITS	Vehicles	Audio	CNN	ACC	2019
[25]	SRS	Vehicles	Smart phone sensors and camera	ANN; RF; KNN	RScore; TScore	2018
[26]	SRS	Vehicles	Camera	KNN	N/A	2018
[27]	SRS	Accident risk	Telemetry	LR; DT; Discriminant analysis; Naive Bayes; SVM; KNN;	ACC	2019
[28]	SRS	Accident risk	Accelerometers	SOM	FPR; Miss detection; Detection delay	2014
[29]	SRS	Pedestrians	Camera	Region-based CNN; SVM; MLP	AUC	2019
[30]	SRS	Pedestrians	Camera	KNN; SVM; ANN; DT	Performance	2018
[31]	SRS	Pedestrian	Camera	HOG based on SVM	Error rate	2017
[32]	SRS	Pedestrian	LIDAR	KNN; Naive Bayes; SVM	Error rate; AUC; Sensitivity; Specificity; Precision; ACC; F1-score	2017
[33]	SRS	Pedestrian	LIDAR	DNN; LSTM; CNN	Classification rate vs. Time to cross; Classification rate vs. Distance to cross	2016
[34]	SRS	Pedestrian	Camera	Haarcascade based on OpenCV library; HOG based on SVM; SSD based on MobileNet; YOLO based on DNN	ACC	2019
[35]	SRS	Animals	Camera; presence sensor	KNN; RF	F1-score	2019
Proposed	SRS	Vehicles	Ultrasound; magnetic field; RADAR	LR; RF; MLP; One-class SVM; LSTM; DRL	TPR; FPR; Precision; ACC; F1-score; AUC	2020

ACC: accuracy; ANN: artificial neural network; AUC: area under the curve; CNN: convolutional neural network; ConvNet: deep convolutional neural network; DNN: dense neural network; DRL: deep reinforcement learning; DT: decision tree; FDR: false discovery rate; FPR: false positive rate; HOG: histogram of oriented gradients; ITS: intelligent transport system; KNN: k-nearest neighbors; logit: logistic regression linear model; LR: logistic regression; LSTM: long short-term memory; MLP: multi-layer perceptron; RBFN: radial basis function network; RF: random forest; SSD: single shot detector; SOM: self-organizing map; SRS: smart road safety; TPR: true positive rate; YOLO: you-only-look-once.
